# White Matter Microstructural Changes and Episodic Memory Disturbances in Late-Onset Bipolar Disorder

**DOI:** 10.3389/fpsyt.2018.00480

**Published:** 2018-10-09

**Authors:** Gilberto Sousa Alves, Christian Knöchel, Michael Anton Paulitsch, Britta Reinke, André F. Carvalho, Richard Feddern, David Prvulovic, Felipe Kenji Sudo, Johannes Pantel, Andreas Reif, Viola Oertel

**Affiliations:** ^1^Institute of General Medicine, Goethe University, Frankfurt/Main, Germany; ^2^Translational Psychiatry Group, Universidade Federal do Ceará, Fortaleza, Brazil; ^3^Laboratory of Neuroscience, Department of Psychiatry, Psychosomatic Medicine and Psychotherapy, Goethe University, Frankfurt/Main, Germany; ^4^Department of Psychiatry, University of Toronto, Toronto, ON, Canada; ^5^Centre for Addiction and Mental Health, Toronto, ON, Canada; ^6^Department of Psychology, Pontifical Catholic University of Rio de Janeiro, Rio de Janeiro, Brazil; ^7^Memory Clinic, D' Or Institute for Research and Education, Rio de Janeiro, Brazil

**Keywords:** DTI, bipolar disorders, TBSS, white-matter, aging, cognition

## Abstract

**Background:** Bipolar disorder (BD) has been associated with distributed network disruption, but little is known on how different clinical subtypes, particularly those with an earlier and later onset of disease, are related to connectivity changes in white matter (WM) tracts.

**Methods:** Diffusion tensor imaging (DTI) and volumetric measures were carried out in early-onset bipolar patients [(EOD) (*n* = 16)], late-onset bipolar disorder [(LOD)(*n* = 14)] and healthy controls (*n* = 32). We also computed ROI analysis of gray matter (GM) and white matter (WM) volumes using the regions with significant group differences in the DTI parameters. Cognitive and behavior measurements were analyzed between groups.

**Results:** Lower fraction of anisotropy (FA) in the right hemisphere comprising anterior thalamic radiation, fornix, posterior cingulate, internal capsule, splenium of corpus callosum was observed in the LOD in comparison with EOD; additionally, lower FA was also found in the LOD in comparison with healthy controls, mostly in the right hemisphere and comprising fibers of the splenium of the corpus callosum, cingulum, superior frontal gyrus and posterior thalamic radiation; LOD also showed worse episodic memory performance than EOD; no statistical significant differences between mood symptoms, WM and GM volumes were found between BD groups.

**Conclusion:** Even after correcting for age differences, LOD was associated with more extensive WM microstructural changes and worse episodic memory performance than EOD; these findings suggest that changes in the WM fiber integrity may be associated with a later presentation of BD, possibly due to mechanisms other than neuroprogression. However, these findings deserve replication in larger, prospective, studies.

## Introduction

Bipolar Disorder (BD) is a chronic condition characterized by marked shifts in mood, energy, activity levels and ability to carry out everyday functions, which affects 1–1.5% of the general population (1, 2). Typically, the onset of the disorder occurs during young adult life, but cases with emergence in late-life have been increasingly reported (3). Compared to Early-onset BD (EOD), subjects with late-onset BD (LOD) were more likely to suffer from depressive symptoms and cognitive difficulties, namely processing speed, executive function and episodic memory impairments, according to observational researches (4, 5).

With the development of modern neuroimaging approaches to assess the neural circuitry in psychiatric disorders, especially the Diffusion Tensor Imaging (DTI), novel information about the microstructural integrity and the directional organization of white-matter (WM) tracts in this population could be obtained (5, 6). For instance, lower fractional anisotropy (FA) was identified in the posterior cingulate, fornix, thalamus, corpus callosum and cerebellum in subjects with BD (7–9). Although substantial evidence indicated that macro and microstructural abnormalities in those networks may be implicated in the pathophysiology of the disorder, current knowledge on structural and functional brain differences between groups with early and late disease-onsets is limited (10, 11). As suggested by a few studies, LOD may present more WM hyperintensities and gray matter (GM) reductions in anterior limbic areas, in comparison to EOD (12, 13), but the association between those features and the clinical manifestations in BD remains obscure (14).

In a previous study from this group, we used tract-based spatial statistics (TBSS) to investigate structural connections between BD and healthy controls. As main results, we have reported reduced fiber integrity and increased mean diffusivity in the fornix, the thalamus and the corpus callosum (both the splenium and the truncus) in BD patients in comparison with controls (10). In the present study, we aimed to explore differences between EOD and LOD through structural volumetry, DTI and clinical parameters using TBSS and voxel-based volumetry (VBM). Our objective was to examine whether cognitive performance in EOD and LOD groups was associated with cortical and WM abnormalities (either global or regional), particularly in the tracts that have been identified as compromised in BD. Based on previous reports (4, 5, 12, 13), we predict that LOD may be associated with more extensive neuronal disruption and higher cognitive impairment in comparison with EOD.

## Methods

### Sample

Methods were described elsewhere (10). Thirty subjects diagnosed with BD type I according to the DSM-IV (15) criteria and thirty-two controls [mean age = 39.22 years [SD = 10.35]] were included in this study. BD group was classified as presenting EOD [age of onset < 27 years; *n* = 16; mean age = 31.12 years [SD = 8.06]] and LOD [age of onset ≥ 27 years; mean age = 48.50 years [SD = 9.63]]. We actually based our group definition on recent evidence presenting a two-component distribution of age of onset (and a sample of 515 BD subjects), with a peak in early adult life and a smaller peak in midlife (16). Parts of the sample have been analyzed in another work by our group [see (10)].

None of the participants were acutely depressed or manic during the procedures, according to the DSM-IV and their scores on the German version of the Beck Depression Inventory (BDI) (scores <18 corresponds to absence of major depression) (17) and the Bech-Rafaelsen Mania Rating Scale (BRMAS) (scores < 7 implies absence of maniac episode) (18). Other exclusion criteria were: (i) history of drug-related disorders; (ii) history of comorbid DSM-IV axis I or II disorder (controls presented no history of any psychiatric disorder); (iii) history of neurological disorder and (iv) inability to provide informed consent. In addition, controls had no family history of affective or psychotic disorder, as demonstrated through the Structured Clinical Interview for DSM-IV (SCID-I and SCID-II; German version) (19).

### Clinical and cognitive assessment

All participants undertook the *Mehrfachwahl-Wortschatz-Intelligenz* test (MWT-B), a measure of general intelligence (20) and the Trail Making A and B, which assess executive function. Episodic memory was tested using the California Verbal Learning Test (CVLT) (21). The following parameters in this test were included: CVLT *discriminability* (CVLT DW), *delayed free recall I* (CVLT DFR 1) and *delayed free recall II* (CVLT DFR 2). Anxiety symptoms were assessed through Strait-Trait Anxiety Inventory (STAI-G) and the Symptom Checklist of Derogatis (SCL 90–R) was used to evaluate global measures of mental and physical health state (22, 23).

### Data acquisition and image processing

All participants undertook MRI using a Trio 3T scanner (Siemens, Erlangen, Germany) with a standard head coil for radiofrequency transmission and signal reception. DTI measures were acquired using an echo planar imaging (EPI) sequence [TR = 8760 ms; TE = 100 ms; bandwith = 1302 Hz/pixel, acquisition voxel size = 2 × 2 × 2 mm^3^; 60 axial adjacent slices; slice thickness = 2 mm (no gap); FOV = 192 × 192 × 120 mm; acquisition matrix = 96 × 96; 10 images without diffusion weighting (b0)] with 60 diffusion-encoded images (b-values = 1000 s/mm^2^ 60 noncolinear directions). Both b0 b 1000 images were averaged three times (total acquisition time = 10 min 31 s). Parallel acquisition of independently constructed images using generalized auto-calibrating parallel acquisitions were used for this sequence (24). This was accompanied by an anatomical scan with each participant (MDEFT; voxel size: 1 × 1 × 1 mm3, 176 slices) (25).

### DTI preprocessing

DTI data were preprocessed and analyzed using the standard procedure of the TBSS software with FSL 4.1 (Oxford Centre for Functional MRI of the Brain - FMRIB software library (FSL, http://www.fmrib.ox.ac.uk/fsl) (26). TBSS is a protocol that employs voxel-wise approach to analyze DTI sequences by projecting individual DTI sequences of each participant on a mean skeleton of a white matter mask (26). The following steps were performed using an in-house script pipeline (MRIST: MR Imaging and Spectroscopy Toolbox, Dept. of Neuroradiology, Oxford University). Each diffusion-weighted volume was affine-aligned to its corresponding b0 image, corrected for potential motion artifacts and eddy-current distortions by rigid transformation to the first image of the merged data set. Following that step, all files were averaged to one single e3D data set for every individual. Subsequently, brain masks of each b0 image with the following parameters were generated: fractional threshold *f* = 0.1, vertical gradient *g* = 0. This was done through FSL's Brain Extraction Tool (BET 2.1; http://www.fmrib.ox.ac.uk/analysis/research/bet/) (27). All subjects' maps were aligned with the most representative subject based on the calculation of the amount of warping needed for the images to be aligned, and further brought in the MNI (Montreal Neurological institute) space by nonlinear registration, using the TBSS routine (26). Finally, a fitting of all corrected images with a tensor model that generated the FA diffusion maps was provided for the TBSS analysis. To average the aligned FA images and create a mean FA image, a FA skeletonization program was used. The skeleton was thresholded at a FA value of 0.2 to limit the effects of poor alignment across subjects and ensure exclusion of CSF and GM voxels. The mean FA image was used to represent the center of all tracts common to the group and comprised all fiber pathways consistently across all participants.

### ROI analysis with VBM

A detailed description of the ROI analysis was published elsewhere (10). The VBM preprocessing and statistical analysis was carried out through the SPM8 (statistical parametric mapping [Wellcome Department of Imaging Neuroscience, London, UK]) and the MATLAB version 7.7.0. All images were assessed and reviewed for artifacts and structural abnormalities. Furtherly, customized T1 templates and prior images of GM, WMm and CSF were created for all participants and were used in the group analysis. Modulated data and prior probability maps (voxel intensity) were employed to guide segmentation in SPM. The segmentation included six different tissue types, light bias regularization (0.001), 60 mm bias FWHM cut-off, warping regularization of 4, affine regularization to the ICBM European brain template (linear registration) and a sampling distance of 3. Before further analysis, the segmentation process was checked for quality. Finally, all images were smoothed (28) with a Gaussian kernel of 8^*^8^*^8 mm^3^ (FWHM), whereby the intensity of each voxel was replaced by the weighted average of the surrounding voxels, blurring the segmented image.

### Statistical analysis

FA maps were computed into general linear modeling (GLM), with covariates including age, education and gender. A global region of interest (ROI) was generated, representing the white matter skeleton and the mean values of FA were drawn. Thereafter, voxel-wise and ROI analysis using TBSS was performed (26). Voxel-wise cross-subject statistics was performed with permutation testing (randomize tool [FSL]). We set the number of permutation to 5,000 as recommended (29). Significance was tested at a *p* < 0.05 level, corrected for multiple comparisons [FWE, family wise error correction]). The voxel-wise analysis was followed by ROI analyses. For this, all significant clusters after voxel-wise analysis (*p* < 0.001) were located using the Jülich Histological [cyto- and myelo-architectonic] Atlas or Harvard-Oxford subcortical structural atlases recommended by FSL to define their topographical boundaries in MNI space. Based on these clusters, white matter masks have been created for each hemisphere. Absolute FA values of the ROIs were extracted from each participant. ANCOVA was applied to control for age differences between subjects in the socio-demographic group comparisons. Scheffe *post-hoc* contrasts were used for group comparisons. Bivariate correlation analysis of GM and WM volume with cognitive parameters and clinical scores was computed with Pearson Product Moment Correlation. Accordingly, bivariate correlation analysis (Pearson Product Moment correlation) between the volume and the medication doses were computed according to the method of Almeida and colleagues (30). The statistical package software SPSS 19.0 was employed for calculations;

### Ethics

Participants were presented a full description of the study and provided written informed consent prior to the enrollment in the study. The protocol was approved by the ethical board of the medical department of the Goethe-University, Frankfurt am Main, Germany. All subjects gave written informed consent in accordance with the Declaration of Helsinki.

## Results

### Sociodemographic characteristics

The social-demographic characteristics of the sample are depicted in the Table 1. LOD subjects were older than controls and EOD (Table 1). Patients with EOD had a mean age of onset of 19 years, while LOD presented the first report of BD with mean age of 40 years. There were no significant differences in education scores between BD groups. Early BD tended to present longer duration of disease and more mood episodes than the LOD group; however, these differences did not reach statistical significance. The use of Lithium (χ2 = 0.153, *df* = 1, *p* = 0.696) or Valproate (χ2 = 0.368, *df* = 1, *p* = 0.544) was not statistically different between EOD and LOD. Furthermore, the dosage of lithium was neither associated with group (*r* = −0.72, *p* = 0.707) nor with age at disease onset (*r* = −0.163, *p* = 0.388).

**Table 1:** Sociodemographic and clinical characteristics and cognitive performance of groups: LOD (*n* = 14), early onset BD (*n* = 16) and the control group (CON; *n* = 32). SD and range are in brackets.

**Variable**	**CON (*n* = 32)**	**Early onset bipolar (*n* = 16)**	**Late onset bipolar (*n* = 14)**	**Significance**

	**Mean (SD)**	**Mean (SD)**	**Mean (SD)**	
Gender	16 f/16 m	7 w/9 m	6 w/8 m	χ2 = 0.28. *p* = 0.87
Age	39.22 (10.36)	31.12 (8.06)	48.50 (9.63)	*F* = 12.06. *p* < 0.01
Years of education	14.86 (2.43)	15.85 (1.84)	15.50 (2.31)	*F* = 1.44, *p* = 0.24
Handedness	All right handed	All right handed		
(L: R)				
Duration of illness	–	11.31 (8.08)	8.86 (5.96)	*F* = 2.43, *p* = 0.36
Number of maniac episodes		10 (12.67)	7.64 (6.15)	F = 0.40, *p* = 0.53
Number of depressive episodes		13 (15.72)	6 (5.44)	*F* = 2.50, *p* = 0.13
Total episodes		23 (6.40)	13.71 (11.04)	*F* = 1.58, *p* = 0.22
Age at disease onset		19.6875 (4.14)	39.78 (7.83)	*F* = 79.99, *p* < 0.001
Age at first psychiatric admission		26.56 (9.47)	40.14 (7.65)	*F* = 18.31, *p* < 0.001
Duration of disease (years)		11.31 (8.08)	8.86 (5.96)	*F* = 0.87, *p* = 0.36
Number of admissions		2.37 (2.19)	4.57 (4.43)	*F* = 3.08, *p* = 0.09
Years of medication therapy		8.79 (8.35)	7.75 (6.41)	*F* = 0.50, *p* = 0.71

*All comparisons by ANOVA, except for gender: χ2 = Chi-Quadrat-Test*.

### Cognitive and clinical assessment

#### EOD vs. LOD

When compared to EOD, LOD subjects significantly performed worse in the CVLT, CVLT-VFW I, and CVLT-VFW-II. Regarding clinical measurements, no differences were found in the severity of current depressive, manic or psychotic symptoms between LOD and EOD groups.

### Volumetric measurement and whole-brain FA maps

**Figure 1:**
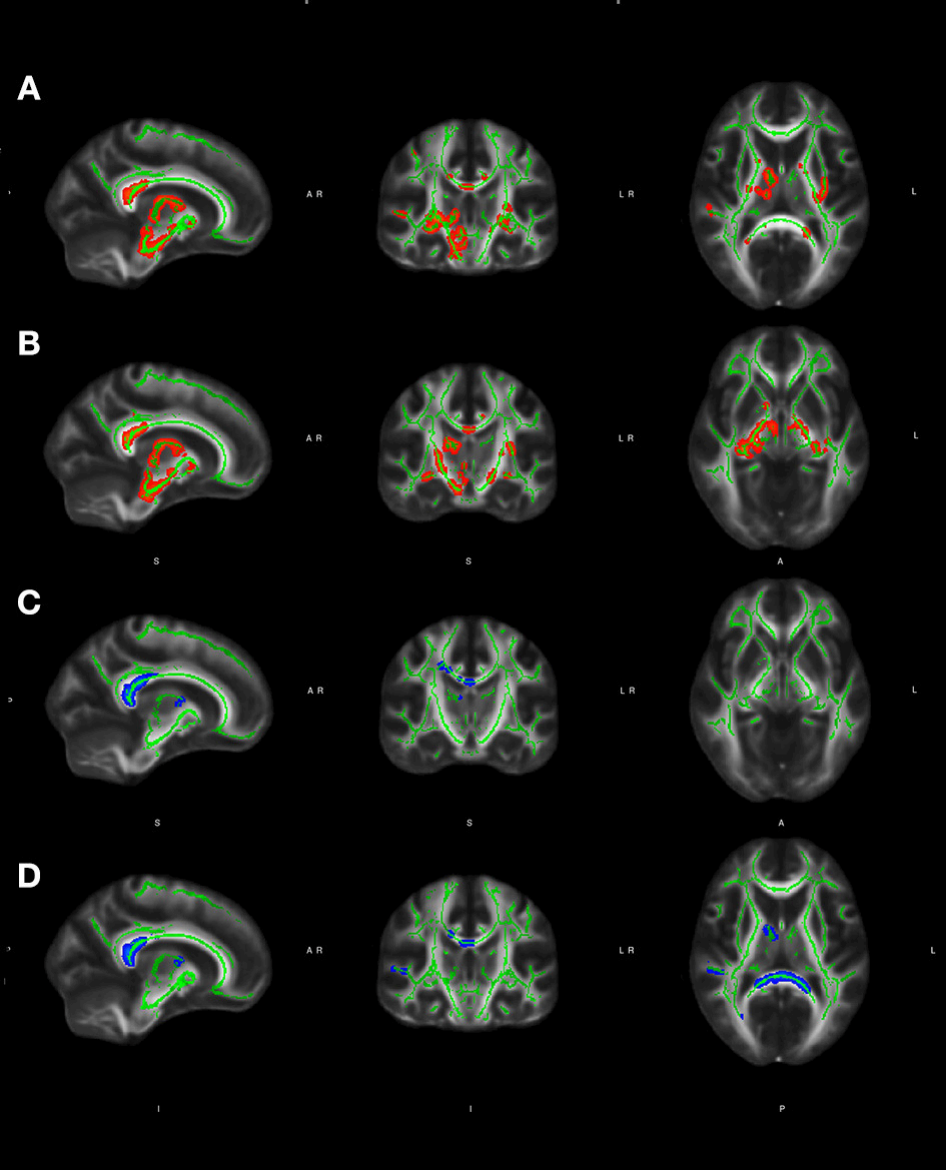
**(A, B)** Significant group effect adjusted for age and gender (thresholded at *p, p* ≤ 0.001) for voxel-wise statistics comparisons (late vs. early onset BD) with whole-brain FA in white matter areas of both hemispheres shown on skeletonised FA maps, using the standard MNI152 1 x 1 x 1 mm brain template; **(C, D)** Significant group effect adjusted for age and gender (thresholded at *p* < 0.001) for voxel-wise statistics comparisons (LOD vs. controls) with whole-brain FA in white matter areas of both hemispheres shown on skeletonised FA maps, using the standard MNI152 1 × 1 × 1 mm brain template. Color-code: red-yellow: early onset BD > LOD, blue-lightblue: controls > LOD. The FA skeleton is marked in green. All images are shown in radiological convention (left is right). Coordinates: **(A)** e **(D)**: X: 10, Y: −8, Z:10; **(B)** e **(C)**: X: 12, Y:−16, Z:4.

Interestingly, no significant differences in GM and WM volumes were found between LOD and EOD in the age-adjusted analysis. Both EOD and LOD exhibited lower WM volume than controls, but a significant statistical significant difference was found only for the EOD-control comparison (Table 2). All whole-brain group comparisons were shown on a mean FA skeleton, using the standard MNI152 1 × 1 × 1 mm brain template, on a *p* ≤ 0.001 significance level (Figure 1, Table 2).

**Table 2:** Volumetric and clinical comparisons between groups—mean (SD) values expressed.

**Test**	**Controls (*n* = 32)**	**EOD (*n* = 16)**	**LOD (*n* = 14)**	***F* ratio**	***P-***value^**^		

	**Mean** ± SD^*^	**Mean** ± SD^*^	**Mean** ± SD^*^		**Con vs. LOD**	**LOD vs. EOD**	**EOD vs. Con**
Gray matter volume	0.368 ± 0.005	0.373 ± 0.008	0.366 ± 0.009	1.68	0.565	0.364	0.568
White matter volume	0.338 ± 0.006	0.363 ± 0.009	0.363 ± 0.010	4.60	0.070	0.731	**0.044**
MWT	31.53 ± 2.58	29.37 ± 3.42	31.07 ± 2.84	3.05	0.883	0.280	0.057
CVLTIs	60.75 ± 1.44	58.05 ± 2.19	50.67 ± 2.44	6.20	**0.001**	**0.042**	0.460
CVLTvfw1	13.14 ± 0.42	12.43 ± 0.63	9.84 ± 0.71	7.85	<**0.001**	**0.014**	0.728
CVLTvfw2	13.56 ± 0.44	13.33 ± 0.66	10.69 ± 0.74	5.42	**0.002**	**0.017**	0.986
TMT A	27.16 ± 7.78	35.69 ± 12.98	38.14 ± 12.95	6.79	**0.007**	0.819	**0.035**
TMT B	56.65 ± 17.41	72.63 ± 28.89	86.64 ± 38.00	6.97	**0.003**	0.349	0.135
TMT total	83.75 ± 22.50	108.31 ± 39.28	119.07 ± 55.25	5.58	**0.013**	0.723	0.090
BDI II	2.48 ± 1.31	13.26 ± 2.08	7.76 ± 2.24	10.6	0.051	0.103	0.001
BRMAS	0.49 ± 0.27	1.17 ± 0.41	0.41 ± 0.46	1.044	0.890	0.258	0.687
SCL 90	0.20 ± 0.07	0.74 ± 0.11	0.469 ± 0.12	9.29	0.065	0.13	0.000
STAI_G	34.12 ± 2.16	35.38 ± 3.00	34.69 ± 3.40	2.782	0.891	0.89	0.116

CVLT, California Verbal Learning Task CVLTIs; BDI II, Beck depression Inventory; BRMAS, Bech Rafaelsen Mania Scale; MWT, Mehrfachwahl-Wortschatz-Test; SCL90, STAI-G: State-Trait Anxiety Inventory; SCL 90, Symptom checklist 90 items; EOD, Early Onset of Disease (< 27 years); LOD, Late Onset of Disease (≥27 years)

* *Estimate Mean*.

** *P = significant on a ≤ 0.05-threshold. Values are highlighted in bold to show significant comparisons at p < 0.05 threshold*.

#### LOD vs. EOD

Voxelwise statistical analysis revealed significantly reduced FA in LOD in comparison with EOD, more extensively on the right hemisphere, in widespread areas comprising the following tracts: splenium of the corpus callosum, fornix (bilateral), cingulum fibers (bilateral), anterior thalamic radiation (right), nuclei of the internal medullary lamina (thalamus), ventroposterior medialis thalamus (right), ventromedial thalamic nucleus (right), pulvinar thalamus (right), anterior limb of the internal capsule (bilateral), retrolenticular part of the internal capsule (bilateral), cerebral penduncle (right), corticospinal tract (right), accumbens nucleus, external capsule (left), midbrain (right), precentral gyrus (right), posterior limb of the internal capsule (bilateral), cerebral peduncle (right), globus pallidus (bilateral).

#### LOD vs. controls

LOD subjects exhibited significantly reduced FA in the posterior cingulate, proportionally distributed on both hemispheres, when compared to healthy controls, in the following tracts: splenium of the corpus callosum (right and left), cingulate (right), superior frontal gyrus (right), corpus callosum frontal (right), anterior thalamic radiation (right), ventroanterior thalamic nucleus (right), superior temporal gyrus (right), posterior thalamic radiation (right).

#### Controls vs. early onset BD

EOB did not show any statistically significant difference when compared to healthy controls.

### Correlations between neuropsychological measurements and FA values

#### LOD vs. EOD

**Figure 2:**
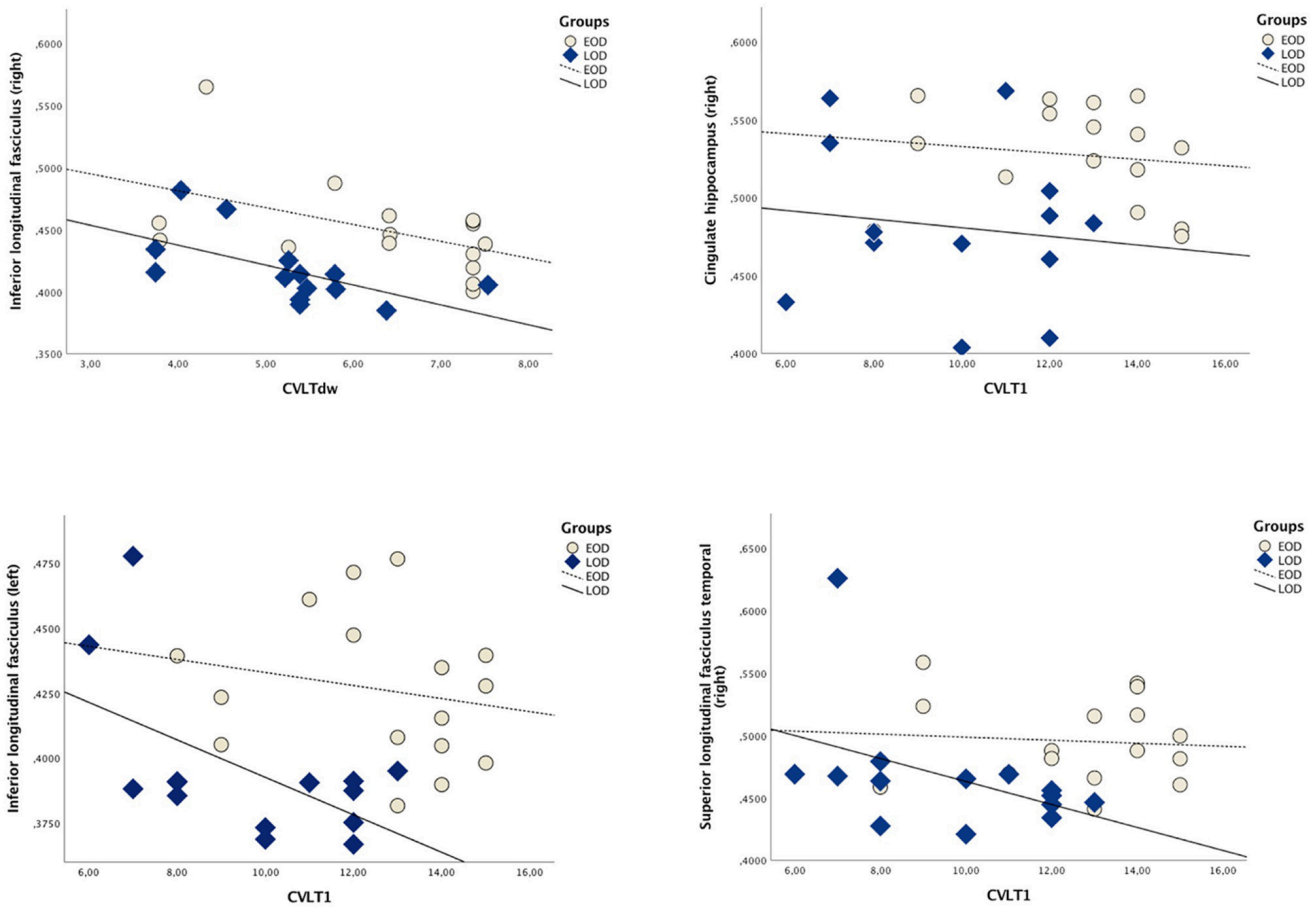
Scatterplot depicting Pearson correlations between WM tracts and CVLT subtests (for correlation values, please refer to the Table 3).

The mean correlations between neuropsychological tests and WM tracts are depicted in the Table 3, Figure 2. Among LOD participants, left inferior longitudinal fasciculus exhibited moderate positive correlations with CVLT1 (*r* = 0.559, *p* = 0.010) and CVLT2 (*r* = 0.540, *p* = 0.010); on the other hand, worse performance on CVLTdw was correlated with higher FA in the right inferior longitudinal fasciculus.

**Table 3:** Pearson rank correlations between FA values and cognitive variables.

**FA**	**TMT A**	**TMT B**	**MWT**	**CVLT total**	**CVLT1**	**CVLT2**	**CVLT DW**
**INFERIOR LONGITUDINAL FASCICULUS (LEFT)**							
Early onset	0.311	0.169	−0.282	−0.235	−0.199	−0.101	−0.215
Late onset	0.020	0.488	0.358	−0.205	−0.559^*^	−0.540^*^	0.062
**INFERIOR LONGITUDINAL FASCICULUS (RIGHT)**							
Early onset	0.130	0.270	0.073	−0.557^*^	−0.460	−0.255	−0.488
Late onset	0.051	0.316	0.165	−0.508	−0.349	−0.432	−0.596^*^
**UNCINATE FASCICULUS LEFT**							
Early onset	0.372	0.151	−0.145	−0.126	−0.157	−0.092	−0.214
Late onset	−0.114	0.463	0.312	−0.177	−0.421	−0.387	−0.328
**UNCINATE FASCICULUS RIGHT**							
Early onset	0.248	0.346	0.184	0.077	0.211	0.280	−0.214
Late onset	−0.516	−0.424	0.550	−0.059	0.039	0.083	−0.007
**SUPERIOR LONGITUDINAL FASCICULUS -TEMPORAL PART (LEFT)**							
Early onset	0.057	−0.034	−0.152	−0.514^*^	−0.609^*^	−0.376	−0.291
Late onset	0.112	0.427	0.270	−0.017	−0.003	−0.005	0.071
**SUPERIOR LONGITUDINAL FASCICULUS -TEMPORAL PART (RIGHT)**							
Early onset	0.120	0.067	0.208	−0.115	−0.080	0.171	0.020
Late onset	0.094	0.453	0.394	−0.117	−0.437	−0.469	−0.426

*FA, fractional anisotropy; MWT, Mehrfachwahl-Wortschatz-Test; CVLT, California Verbal Learning Test; CVLT1, CVLT delayed free recall 1; CVLT 2, CVLT delayed free recall 2; CVLT DW, CVLT discriminality; SLF, Superior Longitudinal Fasciculus; TMT, Trail Making Test*.

* *p < 0.05*.

#### LOD vs. healthy controls

No statistical significant correlations were found. A trend toward significant negative correlation between CVLTdw and FA in the left cingulum hippocampus could be noted (*r* = −489, *p* = 0.076) and MWT showed a trend toward positive correlation with FA in the anterior thalamic radiation left hemisphere (*r* = 0.521, *p* = 0.056).

### Correlations between clinical symptoms and FA values

#### LOD vs. EOD

For LOD individuals, GSI-SCL-90 global scores were moderately correlated with left cingulate hippocampus (*r* = 0.564^*^, *p* = 0.036), right superior longitudinal fasciculus temporal part (*r* = 0.593, *p* = 0.025) and left cingulum fibers (*r* = 0.617, *p* = 0.019). Additionally, Gn-SCL-G90 was negatively correlated with right superior longitudinal fasciculus (*r* = −0.603, *p* = 0.023).

#### LOD vs. healthy controls

Manic symptoms rated by the BRMAS1 correlated positively with FA values in the Forceps Major (*r* = 0.723, *p* = 0.004) and GSI-SCL-90 correlated positively with right superior longitudinal fasciculus temporal part (*r* = 0.571, *p* = 0.033); STAI-G correlated with left cortical spinal tract (*r* = 0.649, *p* = 0.042).

## Discussion

The current study examined WM microstructural integrity in EOD and LOD patients in comparison with healthy controls. Our findings revealed widespread WM changes in the LOD when compared with EOD individuals, particularly in the right hemisphere. No significant abnormalities in diffusion parameters were observed between EOD and healthy controls. Our findings also demonstrated significant cognitive differences between EOD and LOD, particularly in the episodic memory, as observed with CVLT tasks. Contrasting with these results, EOD and LOD did not differ in terms of executive functioning performance. Overall, our study extended the current evidence by showing the presence of several WM abnormalities in late-onset presentation of BD.

The main WM alterations observed in the LOD group comprised the corpus callosum, fornix, cingulum fibers, nucleus accumbens, anterior thalamic radiation, and thalamus, all of them important structures associated with emotional regulation and memory functioning (31–34). The posterior cingulate, for instance, is a key component of the default mode network (DMN), a hub of WM tracts typically showing more activity during rest than in response to external stimulation, for example, during cognitive tasks (35, 36). Disturbances in DMN regions as a possible underlying pathophysiological mechanism in psychiatric diseases like schizophrenia, Alzheimer's disease (AD), and depression (36–38) have been reported more consistently by functional and structural MRI studies. On the other hand, the thalamus, nucleus accumbens and fornix comprise the limbic system, which is regarded as a consistent network associated with BD (39); Thalamic projections, mostly through the fornix, also connect with the hippocampus (40) and microstructural alterations in thalamic fibers have also been reported in several studies with BD (41–45), particularly in the right hemisphere (42, 43, 46). Our findings showing an absence of differences in executive function and worse performance on memory retrieval in the LOD group can also be supported by studies depicting a relatively sparing of working memory and executive functions in BD with later onset, in detriment of processing speed and episodic memory impairment (5, 9). In addition, only minor cognitive differences, particularly in the processing speed, were perceived between EOD and controls. The relatively higher average education of EOD subjects may account for these results, as supported by previous evidence showing higher education as one important moderator decreasing the extent of cognitive impairment between BD and controls (47).

The interpretation of our results in terms of putative mechanisms underlying DTI alterations and the clinical course of BD is puzzling. First, due to the cross-section nature, it remains unclear whether WM abnormalities are cause or consequence of late-onset BD. Secondly, whether WM microstructural changes are the result of primary or second pathological processes (i.e., WM primary degeneration, metabolic or endothelial changes affecting subcortical microvessels, WM degeneration due to GM atrophy) is largely unknown. Although a number of BD studies have linked cognitive decline to volumetric changes, particularly GM reductions (48), the effect these measurements on cognitive or mood variables was considered small in our study, and no significant differences in GM were observed between any group comparison. The absence of macrostructural reductions in LOD relatively to controls was described by Delaloye (5). Taken together, our results suggest that macroscopic volumes changes in WM or GM may not be the core of neurocognitive changes associated with the later onset of BD, as previously suggested (49).

Although we cannot draw any firm conclusions about the neurodegenerative, long-term nature of the observed anatomical changes, the lack of correlation between cognitive variables and age or years of illness speaks against purely progressive neurodegenerative changes due to BD. Indeed, BD itself is associated with complex alterations in cell signaling, neurotransmitters, calcium signaling, which may influence on a synergistic way the ability of cells to deal with extracellular stimuli the way patients respond to environmental events (50). Hence, we may find a better understanding of our findings through alternative hypotheses; the first is the Neuroplasticity model, which addresses the role of reduced connectivity between glial cells and neurons as a putative mechanism affecting synaptic transmission in distributive networks; these neurobiological changes have been reported in the hippocampus and could be associated with the onset and duration of the disease (50, 51) Furthermore, genetic, experimental and lifestyle factor have cumulative effects throughout lifespan (52). Therefore, brain structure and cognitive and behavior changes found in the LOD may both result from poor succeeded compensatory mechanisms, as posited by the Scaffolding model (53). Finally, our findings may also be interpreted according to the Resource-Modulation hypothesis, which assumes that losses in brain structure resource in normal aging (like the case of WM microstructural abnormalities) modulate the effects of common genetic variations, magnifying its effect on cognitive and behavior performance (emotional imbalance) in late adulthood or even late life (54).

## Limitations

As some of the strengths of the current investigation, we assessed a relatively high number of BD patients (*n* = 30) in a remitted state of illness through a multimodal approach, including voxel-based DTI, ROI DTI and VBM of GM; in addition, the high number of directions in the DTI sequence enhanced the validity of results, as suggested by previous studies on the field of BD (45). However, a few limitations need to be addressed. Firstly, all patients have been treated with psychiatric medication during the study investigation. However, we tested the potential influence of psychiatric medication on the structural brain changes and failed to show any associations DTI parameters and medication status or duration of medication. The absence of medication-related structural changes in BD subjects is in agreement with prior studies (8, 12). In despite of that, we were not able to fully control the effect of lithium over the years and thus complete rule out structural changes due to drug therapy. Finally, there is a lack of a consistent definition of EOD and LOD. Indeed, there is no consensual agreement on which age-point should reference the cut off line between EOD and LOD. Different criteria for defining age of onset among LOD, including cut off age of 38.8 (5) and earlier age, around 34.13 (9), have been reported. In despite of the inconsistences on the optimal cut off for defining younger and older subjects, our findings provided additional support on neuroimaging studies using age at onset to identify more homogeneous groups of BD, which may represent distinct subtypes of the disease.

## Concluding remarks

Our study found more widespread WM microstructural in LOD in comparison with early onset BD and controls, with differences being more pronounced in the right hemisphere and in neuronal circuits associated with episodic memory. The significance of such findings is still complex to interpret, but may suggest a closer association between WM microstructural alterations, cognitive impairment and the development of mood changes in older adults. Conversely, the early onset of BD was not associated with either micro or macrostructural age-related brain alterations; this finding represents an evidence against the neurodegeneration hypothesis among early onset BD. While the nature of WM pathological findings in the LOD remains largely unknown, there is a need to clarify whether these findings represent, in its nature, a result of complex pathological processes underpinning BD, particularly those involving the interplay of genetic predisposition, aging, cognitive resilience, regional tract degeneration, and neuronal circuitry depletion. One may also argue whether these WM changes represent a brain endophenotype pointing a higher risk to develop LOD. Future studies with larger samples, follow up measurement and a more universal accepted definition of late and early onset BD will help to clarify these issues.

## Author contributions

GA, VO method design, systematic review of literature and results compilation (including creation of figures and tables), writing of the manuscript (abstract, introduction, methods, results, discussion, limitations and conclusions), selection and organization of bibliographic references; CK, discussion of the theory and method, critical review and text editing; AR, JP, discussion of the theory, critical review, and text editing; FS, discussion of theory, writing of the manuscript, and critical review; AC, discussion of the theory and method, writing of the manuscript, critical review, and text editing; MP, statistical analysis, writing of the manuscript; BR and RF data acquisition, patient selection, statistical analysis; DP, method design, critical review, text editing.

### Conflict of interest statement

The authors declare that the research was conducted in the absence of any commercial or financial relationships that could be construed as a potential conflict of interest.

## Abbreviations

CVLT DFR I: CVLT–delayed free recall I
CVLT DFR II: CVLT–delayed free recall II
CVLT DW: CVLT discriminality
DTI: diffusion tensor imaging
EOD: early onset disorder
LOD: late onset disorder
VM: task performance of verbal episodic memory
STAI-G: Strait-Trait Anxiety Inventory
SCL-90 dep.: SCL-90 d
TMT: Trail Making Test.
